# Association Between the Presence of Multimorbidity and Sociodemographic Factors and Health Care Utilization in the Adult Population of Serbia: A National Health Survey

**DOI:** 10.3390/healthcare14152371

**Published:** 2026-08-03

**Authors:** Ognjen M. Djordjevic, Svetlana R. Radevic, Snezana M. Radovanovic, Olgica B. Mihaljevic, Milos M. Stepovic, Jovana Z. Radovanovic Selakovic, Irfan F. Corovic, Natasa K. Rancic, Gordana V. Djordjevic

**Affiliations:** 1 Department of Epidemiology, Faculty of Medical Sciences, University of Kragujevac, 34000 Kragujevac, Serbia; ognjendjordjevic763@gmail.com (O.M.D.); jovana.070698@gmail.com (J.Z.R.S.); mogidj@ptt.rs (G.V.D.); 2 Institute of Public Health Kragujevac, 34000 Kragujevac, Serbia; cecaradevic@yahoo.com (S.R.R.); jovanarad@yahoo.com (S.M.R.); 3 Department of Social Medicine, Faculty of Medical Sciences, University of Kragujevac, 34000 Kragujevac, Serbia; 4 Department of Pathophysiology, Faculty of Medical Sciences, University of Kragujevac, 34000 Kragujevac, Serbia; vrndic07@yahoo.com; 5 Department of Anatomy, Faculty of Medical Sciences, University of Kragujevac, 34000 Kragujevac, Serbia; stepovicmilos@yahoo.com; 6 University Clinical Center Kragujevac, 34000 Kragujevac, Serbia; 7 Center for Molecular Medicine and Stem Cell Research, Faculty of Medical Sciences, University of Kragujevac, 34000 Kragujevac, Serbia; ira.corovic@gmail.com; 8 Department of Internal Medicine, General Hospital of Novi Pazar, 36300 Novi Pazar, Serbia; 9 Faculty of Medicine Nis, University of Nis, 18000 Nis, Serbia; 10 Center for Diseases Control and Prevention, Institute for Public Health Nis, 18000 Nis, Serbia

**Keywords:** multimorbidity, health care utilization, socioeconomic factors, National Health Survey, Serbia

## Abstract

Introduction: Multimorbidity, defined as the coexistence of two or more chronic conditions in the same person, represents a major challenge for modern health systems due to its association with adverse health outcomes, increased mortality, and high healthcare utilization. We aimed to assess the prevalence of multimorbidity in the adult population of Serbia and examine its association with demographic and socioeconomic factors and healthcare utilization. Methodology: Data were drawn from the 2019 Serbian National Health Survey. Descriptive statistics, chi-square and independent-samples *t*-tests, as well as univariate and multivariate logistic regression analyses, were performed to examine the associations. Results: The study included 12,439 adults—48.5% men and 51.5% women (mean age: 52.83 ± 17.69). Multimorbidity was present in 35.2% of participants. It was significantly more frequent among women than men (39.65% vs. 30.53%, *p* < 0.001) and increased markedly with age, reaching 68.63% among individuals aged ≥80 years. Participants with multimorbidity were significantly older than those without it (*p* < 0.001). Higher prevalence was observed among widowed individuals, participants with lower education, economically inactive persons, those with poorer material status, and residents of Southern and Eastern Serbia (all *p* < 0.001). Multivariable regression analysis confirmed independent associations between multimorbidity and female sex, older age, lower education, poorer socioeconomic status, and regional disparities. Multimorbidity was a strong predictor of healthcare utilization. Individuals with multimorbidity were significantly more likely to use inpatient and day hospital services, visit specialist physicians, use prescribed medications, and receive home care (all *p* < 0.05). Conclusion: Multimorbidity affects more than one-third of adults in Serbia and is strongly associated with demographic and socioeconomic disadvantage. It substantially increases healthcare utilization, underscoring the need for integrated, patient-centered care and targeted public health interventions.

## 1. Introduction

Defined as the concurrent presence of two or more chronic diseases or conditions in the same individual, multimorbidity is recognized as one of the major challenges facing contemporary medicine and health care systems [1] and has become a major global public health challenge. Multimorbidity is associated with substantial adverse consequences, including increased vulnerability to acute illness, worsening of existing chronic diseases, more frequent hospitalizations, and higher health care expenditures. Previous studies have shown that approximately one in two adults aged 65 years and older in the United States of America (USA) are affected by multimorbidity [2]. The prevalence of multimorbidity has increased over the past two decades but has remained relatively stable since 2010, suggesting that progress in reducing its overall burden has been limited. Approximately one-half of the adult population in South America is affected by multimorbidity, and, based on the findings of Chowdhury et al. (2023), the highest prevalence of multimorbidity was observed in South America, whereas Africa showed the lowest prevalence [1]. Multimorbidity is highly prevalent in both China and India, with median prevalence estimates of 36.1% and 28.3%, respectively [3]. Based on the results reported by Gomes Dantas et al. (2026), who conducted a large cross-sectional study including 68,593 participants aged 50 years and older from 27 European countries and Israel, the overall prevalence of multimorbidity was 37.9% [4]. Multimorbidity was significantly associated with female sex, older age, low educational level and tobacco and alcohol consumption. A higher prevalence of multimorbidity was also observed among individuals with poor self-rated health, obesity, functional limitations, severe loneliness, and lower quality of life. These findings further emphasize the important role of sociodemographic, behavioral, and health-related factors in the development and distribution of multimorbidity among older adults [5].

Patients with multimorbidity have more complex health needs, a higher likelihood of adverse health outcomes, lower quality of life, and an increased risk of mortality [4]. Multimorbidity represents one of the most significant challenges of modern medicine and public health at the global level. In recent decades, driven by increased life expectancy and the epidemiological transition toward noncommunicable disease predominance, health systems worldwide have been confronted with a growing number of individuals living with multiple chronic conditions simultaneously [4]. Despite its higher prevalence among older adults, as consistently demonstrated by studies conducted across Europe, North America, and other regions [5], multimorbidity is not exclusively an age-related phenomenon but is also strongly associated with socioeconomic status, educational attainment, living conditions, and health-related behaviors [6]. Pathirana and Jackson demonstrated that lower socioeconomic status is consistently associated with a higher prevalence of multimorbidity. Their findings indicate that individuals with lower income, lower educational attainment, and greater socioeconomic deprivation are significantly more likely to experience multimorbidity than those with more favorable socioeconomic circumstances [7]. This phenomenon is increasingly examined in the international literature through the lens of health inequalities and the concept of social determinants of health, which emphasize that an individual’s health status is shaped not only by biological factors but also by the broader social context [7]. In addition to its impact on quality of life and functional capacity, multimorbidity imposes a substantial burden on health systems, being associated with increased use of health services, a higher number of physician visits, more frequent hospitalizations, polypharmacy, and greater direct and indirect treatment costs [5,8]. Contemporary research within the European Union (EU) suggests that traditionally organized, disease-specific models of health care are often inadequate for managing patients with multiple chronic conditions, highlighting the need for integrated, patient-centered approaches [9]. In this context, analyzing the relationship between multimorbidity and patterns of health care utilization is of particular importance, enabling an assessment of the actual burden on the system and the identification of groups with the greatest needs [10].

In the Republic of Serbia, characterized by pronounced population aging and specific socioeconomic challenges typical of countries in transition, multimorbidity has gained additional significance [11]. According to data from the Institute of Public Health of Serbia “Dr Milan Jovanović Batut,” chronic noncommunicable diseases are the leading causes of morbidity and mortality, indicating a substantial potential comorbidity burden in the adult population [12]. Socioeconomic inequalities, regional disparities in development and access to health services, as well as differences in health-related behaviors, may influence the occurrence and progression of multiple chronic conditions. Individuals with multimorbidity in Serbia are likely to use primary health care services, specialist consultations, and hospital care more frequently, with important implications for the organization and sustainability of the health system [13]. National health surveys represent a reliable source of data for comprehensive analyses of the prevalence and determinants of multimorbidity, as they enable the simultaneous assessment of health indicators, demographic characteristics, and socioeconomic factors in representative samples [14]. Through the analysis of data from the National Health Survey in Serbia, it is possible to investigate the association between multimorbidity and age, sex, level of education, employment status, and material well-being, as well as to evaluate the impact of multiple chronic diseases on the intensity and structure of health care utilization [15]. By placing the findings within a broader European and global context, this study aims to contribute to the international literature on multimorbidity, particularly from the perspective of a middle-income country with specific demographic and social patterns. Understanding these relationships is essential for designing targeted public health interventions, improving integrated health care, and reducing health inequalities, both in Serbia and in the wider European context.

We aimed to determine the prevalence of multimorbidity in the adult population of the Republic of Serbia, as well as to examine the associations of demographic and socioeconomic factors and health care utilization with multimorbidity.

## 2. Material and Methods

### 2.1. Study Design

This descriptive, analytical, cross-sectional study based on a representative sample of the Serbian population is part of the 2019 National Health Survey of Serbia, conducted by the Statistical Office of the Republic of Serbia in cooperation with the Institute of Public Health “Dr Milan Jovanović Batut” and the Ministry of Health. The target population comprised any person aged 20 and over living in private households, representing the general population.

The inclusion criteria:

- all adults older than 20 years who gave their informed consent.

The exclusion criteria were:

- people under the age of 20,
- incomplete questionnaires,
- people with health problems preventing them from being able to stand up,
- individuals were also excluded if they lived in institutions or collective accommodation (e.g., student dormitories, nursing homes, psychiatric institutions, prisons, or monasteries),
- were illiterate, could not understand the ethical principles of participation, or
- were physically or mentally incapable of participating.

### 2.2. Sample

The 2019 National Health Survey of Serbia used a nationally representative, stratified, two-stage random sample. Stratification was based on settlement type (urban and other) and geographic region (Belgrade, Vojvodina, Šumadija and Western Serbia, Southern and Eastern Serbia), using the 2011 Census as the sampling frame. Census tracts served as primary sampling units and were selected proportionally to the number of households, followed by random household selection. Non-response at the household and individual levels was addressed by adjusting weights, ensuring that estimates remained representative of the non-institutionalized adult population. Missing data in the analyses were handled using a complete-case approach, including only respondents with available data for all variables relevant to the given analysis. This is in line with the recommendations of the European Health Interview Survey (EHIS Wave 3) and preserves the statistical validity of the results, given the relatively low proportion of missing values in the dataset.

Data were collected from October to December 2019 in accordance with the guidelines of the European Health Interview Survey (EHIS) Wave 3. Participation was voluntary, with informed consent from all respondents. Privacy and confidentiality were strictly protected by anonymization, secure data storage and removal of personal identifiers, ensuring that no individual could be identified in the published results.

The sample size was calculated based on EUROSTAT precision requirements for the indicator “proportion of persons limited in performing daily activities” [16]. Assuming an 8% prevalence, with a desired error below 1% and 95% confidence, approximately 6000 households (~15,000 individuals) were needed to ensure nationally and regionally representative estimates. Ten households per enumeration area across 600 areas were selected, with reserves for non-response.

Data collection adhered to EHIS Wave 3 methodology over three months (October–December 2019), in line with recommendations that fieldwork cover at least three months including autumn, in accordance with the official methodological guidelines issued by Eurostat [15]. Personally identifiable data were coded and stored securely, and the results were reported in an aggregated form. The dataset was provided to the University of Kragujevac for research purposes.

#### Ethical Considerations

The study was conducted under the institutional ethics approval issued to the Faculty by the Ethics Committee of the Institute of Public Health of Serbia (Approval No. 7703/1, issued on 8 December 2021). The institution does not issue individual researcher-specific stamped or signed certificates; instead, ethical approval is recorded and maintained at the Faculty level. Additionally, the ethical standards of the 2019 Serbian National Health Survey were fully aligned with the Declaration of Helsinki (originally adopted in 1964 and last amended in 2013) and with national legislation. The survey followed all regulations related to the implementation of the third wave of EHIS, based on the Commission Implementing Regulation (EU) No. 255/20184.

Measures for ensuring privacy and confidentiality were undertaken in accordance with the General Data Protection Regulation (GDPR), the national Personal Data Protection Law, the Personal Data Protection Strategy (Official Gazette of the Republic of Serbia, No. 87/2018), the Law on Official Statistics, the Decision on the Official Statistics Program for the Period 2016–2020 (“Official Gazette of RS”, No. 55/2015) and the Regulation on the 2019 Official Statistics Plan (“Official Gazette of RS”, No. 105/201).

Participants received written information about the study purpose, rights, and contact details for questions or complaints. Informed consent was obtained in writing from all participants. Anonymity was ensured by removing any identifying information and replacing it with coded data. All databases are stored on protected servers, and results are published exclusively in aggregated form, ensuring full confidentiality of individual data.

### 2.3. Questionnaires

In this study, we used standardized questionnaires based on the European Health Interview Survey (EHIS, Wave 3), adapted to the local context, with a single measurement form [16]. Three instruments were used: a household information panel to collect data on all household members and the household’s socioeconomic profile, face-to-face interviews, and self-administered questionnaires. The independent variables in this study are as follows: demographic variables (age and gender structure, marital status, type of settlement, region); socioeconomic variables (employment status, educational structure, Well-being Index as an indicator of the material condition of the respondents, self-rated health); and aspects of health care utilization, including inpatient and outpatient health care, home care and treatment services, emergency medical care, medication use, preventive services, use of private practice services, and unmet health care needs.

The presence of chronic disease was analyzed based on the respondents’ self-reporting of whether they had been diagnosed by a doctor with any of the following diseases:

1. Asthma (including allergic asthma);
2. Chronic bronchitis, chronic obstructive pulmonary disease, or emphysema;
3. Myocardial infarction (heart attack) or chronic consequences of myocardial infarction;
4. Coronary heart disease or angina pectoris;
5. High blood pressure (hypertension);
6. Stroke (cerebral hemorrhage, cerebral thrombosis—stroke) or chronic consequences of stroke;
7. Arthrosis—degenerative joint disease (not including arthritis—inflammation of the joints);
8. Lower spine deformity or other chronic back problem;
9. Cervical deformity or other chronic cervical spine problem;
10. Diabetes (diabetes);
11. Allergies such as allergic rhinitis, hay fever, conjunctivitis, dermatitis, food allergy or other;
12. Allergies (not including asthma);
13. Cirrhosis of the liver;
14. Inability to hold urine (urinary incontinence) and problems controlling the bladder;
15. Kidney problems;
16. Depression;
17. Cancer (malignant disease);
18. Increased blood fat (cholesterol);
19. Obesity (Body Mass Index—BMI ≥ 30 kg/m^2^).
20. The dependent variable in the study was multimorbidity, defined as the concurrent presence of two or more chronic diseases or conditions [17].

### 2.4. Statistical Analysis

To analyse factors influencing multimorbidity in the Serbian population, we first proceeded with a bivariate analysis. For qualitative variables, the Chi-square (χ^2^) test was used to compare differences between groups. For quantitative variables, Student’s *t*-test was used after verifying the normality of the distribution. We calculated the Odds Ratios (ORs) and their 95% confidence intervals (95% CIs). For multivariable analysis, we included variables with *p* < 0.20 in the bivariate analysis in the model using logistic regression. Significance was set at *p* < 0.005. All statistical calculations were performed using the commercial standard software package SPSS version 20.0.

## 3. Results

In this study, we included 12,439 participants, of whom 6032 (48.5%) were men and 6407 (51.5%) were women, with a mean age of 52.83 ± 17.69 years (range 20–99). The mean age of the men was 51.73 ± 17.51 years (range 20–98), while the mean age of the women was 53.87 ± 17.81 years (range 20–99) (Table 1).

The demographic and socioeconomic characteristics of participants with multimorbidity are presented in Table 1.

The highest prevalence was observed in the oldest age groups, particularly among participants aged 80 years and older (68.63%), followed by those aged 70–79 years (66.28%) and 60–69 years (54.92%) (*p* < 0.001). Furthermore, the mean age of participants with multimorbidity was significantly higher than that of those without (64.39 ± 13.14 vs. 46.54 ± 16.65 years; independent-samples *t*-test, *p* < 0.001). Regarding marital status, the highest prevalence of multimorbidity was recorded among widowed participants (64%, *p* < 0.001). Multimorbidity was also more prevalent among participants from the regions of Southern and Eastern Serbia (40.27%, *p* < 0.001), those with primary or lower levels of education (55.05%, *p* < 0.001), economically inactive individuals (55.66%, *p* < 0.001), and participants belonging to the poor and poorest socioeconomic categories (39.11%, *p* < 0.001).

Of the total study population, 5703 participants (45.8%) had no chronic disease, 2353 (18.9%) had a single diagnosed chronic condition, and 4383 (35.2%) had multimorbidity (Table 2). Multimorbidity was detected in 4383 (35.2%) participants, and the presence of a single disease was detected in 2353 (18.9%) participants, while the remaining 5703 (45.8%) had no multimorbidity (Table 1). Multimorbidity was more frequent in women compared with men (39.65% vs. 30.53%, *p* < 0.001).

The distribution of health care utilization according to the presence of multimorbidity is presented in Table 3.

Among participants who had used inpatient hospital care in the previous 12 months, 64.8% were individuals with multimorbidity, indicating a statistically significant difference in the frequency of hospital care utilization between participants with and without multimorbidity (*p* < 0.001). Similarly, 62.8% of participants with multimorbidity reported using day hospital services, demonstrating that multimorbidity significantly influences the utilization of day hospital care (*p* < 0.001) (Table 3). Among users of medications not prescribed by a physician, there was a slightly higher proportion of participants without multimorbidity compared with those with multimorbidity (55.2% vs. 44.8%). However, statistically significant differences were observed both among participants who did not use non-prescribed medications (χ^2^ = 267.85, *p* < 0.001) and in relation to prescribed medications. Specifically, the proportion of participants with multimorbidity was nearly twice as high among those using physician-prescribed medications compared with participants without multimorbidity (61.8% vs. 38.2%, *p* < 0.001). The proportion of participants with multimorbidity who reported having a chosen physician in the public sector (37.6%) or a physician in private practice (34.5%) was significantly lower compared with participants without multimorbidity (62.4% for a public sector physician and 65.5% for a private sector physician). A considerably higher proportion of participants without multimorbidity reported having a gynecologist, both in the public sector (62.9% vs. 37.1%) and in private practice (76.4% vs. 23.6%), compared with participants with multimorbidity. Only slightly more than one-quarter of participants (27.8%) who reported having and visiting a dentist in private practice were individuals with multimorbidity. A significantly higher frequency of visits to physiotherapists (63.4%) and psychiatrists/psychologists (60.5%) was observed among participants with multimorbidity. Overall, individuals with multimorbidity used private health care services significantly less frequently than those without multimorbidity. In contrast, the proportion of participants using home care services was significantly higher among individuals with multimorbidity (67.6%) (Table 3).

The results of the bivariate analysis and multivariate regression models examining the association between demographic and socioeconomic characteristics and multimorbidity are presented in Table 4.

The bivariate analysis showed that the risk of multimorbidity was 1.49 times higher among women (OR = 1.49). Multimorbidity was 2.86 times more frequent among widowed participants (OR = 2.86), 1.36 times more frequent among participants from Southern and Eastern Serbia (OR = 1.36), 3.49 times more frequent among participants with primary or lower education (OR = 3.49), and 1.20 times more frequent among those with secondary education (OR = 1.20). Additionally, multimorbidity was 4.58 times more frequent among employed participants (OR = 4.58) and 1.44 times more frequent among participants with poorer material status (OR = 1.44). With respect to age, younger age groups had a significantly lower risk of multimorbidity. The multivariate regression analysis largely confirmed the findings of the univariate analysis (Table 4).

The results of the univariate and multivariate regression models examining the association between health care utilization and multimorbidity are presented in Table 4.

Participants with multimorbidity demonstrated a substantially higher likelihood of using health care services. In the bivariate analysis, individuals with multimorbidity were 10.89 times more likely to visit a specialist physician within the previous 12 months (OR = 10.89), 7.63 times more likely to use physician-prescribed medications (OR = 7.63), and 3.95 times more likely to use home care services in the previous 12 months (OR = 3.95). The multivariate regression analysis confirmed these associations, indicating that multimorbidity is a strong independent predictor of increased health care utilization (Table 5).

## 4. Discussion

The results of this nationally representative study indicate that multimorbidity affects more than one-third of the adult population in Serbia (35.2%), placing Serbia within the prevalence range reported in contemporary European and global studies. In recent studies, the prevalence of multimorbidity in the general adult population typically ranges between 30% and 40%, with a marked increase with age and pronounced socioeconomic gradients [13,14]. European population analyses show that countries with aging populations and a high burden of non-communicable diseases display prevalence patterns similar to those observed in our study, suggesting that Serbia does not differ in overall prevalence but rather in the specific distribution of risk factors and patterns of healthcare utilization [18]. One of the most consistent findings in the current literature is the strong association between multimorbidity and age [19], which was confirmed in our study. The prevalence of 68.63% among individuals aged 80 years and older and over 66% in the 70–79-year age group aligns with European cohort studies showing that most older adults live with at least two chronic conditions [20,21]. The mean age of participants with multimorbidity was nearly 18 years higher than that of participants without multimorbidity, reflecting the cumulative effect of prolonged exposure to risk factors, biological aging, chronic inflammation, and gradual loss of physiological reserve. However, recent studies also highlight that multimorbidity increasingly occurs in middle age, particularly among socioeconomically disadvantaged groups, suggesting that age interacts with social determinants of health [22,23].

The substantially higher prevalence of multimorbidity among women (39.65% vs. 30.53%) is consistent with numerous publications indicating that women are more likely to report chronic conditions, have a longer life expectancy, and are more likely to accumulate multiple diagnoses [24,25]. Potential explanations include biological differences, greater healthcare-seeking behaviour among women, higher diagnostic detection, and the fact that women tend to live longer with chronic conditions that are not necessarily fatal but significantly affect quality of life. The literature also emphasizes the impact of gendered social roles, chronic stress, and economic dependency in older age, which may further increase the risk of disease accumulation [26].

The pronounced socioeconomic gradient observed in our study represents a key finding. Higher rates of multimorbidity among participants with lower education, poorer material status, and inactive employment are in line with contemporary European and global studies showing that individuals of lower socioeconomic status not only have a higher disease burden but also develop multiple chronic conditions earlier in life [6,27]. Education functions as a critical structural determinant by influencing health literacy, access to information, lifestyle, and employment opportunities. Poorer material status is associated with greater exposure to hazardous working conditions, an unhealthy diet, limited access to preventive services, and chronic psychosocial stress, all of which increase the likelihood of developing multimorbidity [27]. The finding of higher risk among participants from Southern and Eastern Serbia may reflect regional socioeconomic inequalities, differences in healthcare availability, and variations in demographic composition.

The marital status findings are particularly notable, with widowed individuals having a nearly three times higher risk of multimorbidity. Current literature indicates that the loss of a partner constitutes a major psychosocial stressor that can worsen existing chronic conditions and reduce self-care [28]. Additionally, widowhood is more common in older age groups, which further amplifies the risk [29]. Findings related to healthcare utilization clearly demonstrate that multimorbidity substantially increases the burden on the healthcare system. More than two-thirds of hospital and day-care service users had multimorbidity, while univariate analyses showed that these individuals were almost eleven times more likely to visit a specialist and seven times more likely to use prescribed medication. These results are consistent with international studies showing that multimorbidity is one of the strongest predictors of frequent hospitalization, polypharmacy, and fragmented care [29]. The higher use of home care and physiotherapy among individuals with multimorbidity likely reflects the need for long-term support and management of functional limitations.

An interesting finding is that individuals with multimorbidity were less likely to use private healthcare services, which may be explained by economic constraints, as these individuals are more often in lower-income groups. Current research indicates that patients with a higher disease burden rely more heavily on the public healthcare system, which may increase public resource use and exacerbate inequalities in access to high-quality, continuous care [30,31].

The multivariable analysis, which confirmed the independent association of multimorbidity with demographic and socioeconomic factors, as well as increased healthcare utilization, indicates that multimorbidity is a complex, multifactorial phenomenon. Our findings support contemporary concepts of integrated healthcare, emphasizing the need for a holistic, patient-centred approach, coordination across levels of care, and strengthening primary care as a key point for managing chronic conditions [31,32]. The management of multimorbidity requires a person-centred, integrated approach based on comprehensive assessment, continuity of care, and coordination of services, with primary care playing a central role in care delivery [33].

Overall, the results of this study are consistent with contemporary international evidence while also highlighting specific characteristics of the Serbian context, including pronounced regional and socioeconomic inequalities. These findings underscore the need for targeted public health interventions aimed at early detection and prevention of chronic diseases among vulnerable groups, as well as the reorganization of healthcare services toward integrated, continuous, patient-centred models of care, which represent one of the key challenges for modern healthcare systems in Europe and beyond.

This study has certain limitations that should be considered. The cross-sectional design does not allow the determination of causal relationships but only associations between variables. Data on chronic conditions and healthcare utilization were based on self-report, which may lead to information bias and under- or overestimation of certain conditions. The definition of multimorbidity did not include disease severity or specific disease clusters, and residual confounding factors may have influenced the analysis. Nonetheless, the national representativeness of the sample and the scope of the data constitute a major strength of this study.

## 5. Conclusions

Multimorbidity affects more than one-third of the adult population in Serbia and is strongly associated with age, female sex, lower education, poor material status, widowhood, and regional inequalities. Individuals with multimorbidity utilize specialist, hospital, and home care services, as well as prescribed medications, significantly more frequently, indicating a substantial burden on the healthcare system. These findings emphasize the need for early detection of chronic conditions, strengthening preventive programs targeting socioeconomically vulnerable groups, and developing integrated, patient-centred healthcare models with a stronger role for primary care. Strategic resource planning and the reduction of health inequalities represent key steps in addressing the growing burden of multimorbidity in Serbia.

## Figures and Tables

**Table 1:** Demographic and socioeconomic characteristics of participants with multimorbidity.

Variable	Category	Total (N = 12,439)	Absence of Multimorbidity (N = 8056)	Presence of Multimorbidity (N = 4383)	*p* *
Sex	Female	6407 (51.5%)	3866 (60.35%)	2541 (39.65%)	<0.001
	Male	6032 (48.5%)	4190 (69.47%)	1842 (30.53%)	
Age group (years)	20–29	1545 (12.4%)	1493 (96.63%)	52 (3.37%)	<0.001
	30–39	1762 (14.2%)	1603 (90.97%)	159 (9.03%)	
	40–49	1981 (15.9%)	1610 (81.27%)	371 (18.73%)	
	50–59	2215 (17.8%)	1414 (63.83%)	801 (36.17%)	
	60–69	2551 (20.5%)	1150 (45.08%)	1401 (54.92%)	
	70–79	1604 (12.9%)	541 (33.72%)	1063 (66.28%)	
	80+	781 (6.3%)	245 (31.37%)	536 (68.63%)	
Mean age	X ± SD	52.83 ± 17.69 years (min 20, max 99)	46.54 ± 16.65	64.39 ± 13.14	<0.001 **
Marital status	Single	2265 (18.0%)	2049 (90.46%)	216 (9.54%)	<0.001
	Married	7844 (63.1%)	4999 (63.73%)	2845 (36.27%)	
	Widowed	1672 (13.4%)	602 (36.0%)	1070 (64.0%)	
	Divorced	658 (5.3%)	406 (61.7%)	252 (38.3%)	
Region	Vojvodina	2793 (22.5%)	1719 (61.54%)	1074 (38.46%)	<0.001
	Šumadija and Central Serbia	3977 (32.0%)	2742 (68.94%)	1235 (31.06%)	
	Southern and Eastern Serbia	2757 (22.2%)	1647 (59.73%)	1110 (40.27%)	
	Belgrade	2912 (23.4%)	1948 (66.89%)	964 (33.11%)	
Education level	Primary or lower	3070 (24.7%)	1380 (44.95%)	1690 (55.05%)	<0.001
	Secondary	7009 (56.3%)	4928 (70.31%)	2081 (29.69%)	
	Higher/University	2352 (18.9%)	1742 (74.06%)	610 (25.94%)	
Employment status	Unemployed	2293 (18.3%)	1745 (76.10%)	548 (23.90%)	<0.001
	Employed	4648 (37.4%)	3827 (82.33%)	821 (17.67%)	
	Economically inactive	5.363 (43.1%)	2378 (44.34%)	2985 (55.66%)	
Material status	Poor/Poorest	5.022 (40.4%)	3.058 (60.89%)	1.964 (39.11%)	<0.001
	Middle class	2.525 (20.3%)	1.615 (63.96%)	910 (36.04%)	
	Rich/Richest	4.892 (39.3%)	3.383 (69.15%)	1.509 (30.85%)	
Self-rated health	Poor/Very poor	1.450 (11.7%)	617 (84.5%)	1.129 (15.5%)	<0.001
	Average	3.025 (24.3%)	1.186 (39.2%)	1.839 (60.8%)	
	Good/Very good	7.302 (58.7%)	234 (16.1%)	1.216 (83.9%)	

* Chi-square test; ** independent-samples *t*-test.

**Table 2:** Distribution of chronic diseases in the study population according to gender.

Chronic Disease	Total N (%)	Men N (%)	Women N (%)	*p*
Asthma (including allergic asthma)	477 (3.8)	219 (3.63)	258 (4.02)	*p* = 0.262
Chronic bronchitis, chronic obstructive pulmonary disease, or emphysema	464 (3.7)	207 (3.43)	257 (4.01)	*p* = 0.098
Myocardial infarction (heart attack) or chronic consequences of myocardial infarction	267 (2.1)	182 (3.02)	85 (1.32)	*p* < 0.001
Coronary heart disease or angina pectoris	1293 (10.4)	556 (9.21)	737 (11.50)	*p* < 0.001
High blood pressure (hypertension)	4259 (34.2)	1883 (31.21)	2376 (37.08)	*p* < 0.001
Stroke (cerebral hemorrhage, cerebral thrombosis—stroke) or chronic consequences of stroke	185 (1.5)	110 (1.82)	75 (1.17)	*p* = 0.003
Arthrosis—degenerative joint disease (not including arthritis—inflammation of the joints)	1019 (8.2)	306 (5.07)	713 (11.13)	*p* < 0.001
Lower spine deformity or other chronic back problem	2426 (19.5)	956 (15.85)	1470 (22.94)	*p* < 0.001
Cervical deformity or other chronic cervical spine problem	1714 (13.8)	581 (9.63)	1133 (17.68)	*p* < 0.001
Diabetes (diabetes)	1138 (9.1)	537 (8.90)	601 (9.38)	*p* = 0.183
Allergy such as allergic rhinitis, hay fever, conjunctivitis, dermatitis, food allergy or other allergies (not including asthma)	888 (7.1)	333 (5.52)	555 (8.66)	*p* < 0.001
Cirrhosis of the liver	43 (0.3)	22 (0.36)	21 (0.33)	*p* = 0.421
Inability to hold urine (urinary incontinence) and problems controlling the bladder	513 (4.1)	256 (4.24)	257 (4.01)	*p* = 0.528
Kidney problems	547 (4.4)	230 (3.81)	317 (4.94)	*p* = 0.002
Depression	612 (4.9)	226 (3.74)	386 (6.02)	*p* < 0.001
Cancer (malignant disease)	1483 (11.9)	566 (9.38)	917 (14.31)	*p* < 0.001
Increased blood fat (cholesterol)	248 (2.0)	93 (1.54)	155 (2.42)	*p* = 0.001
Obesity (Body Mass Index—BMI ≥ 30 kg/m^2^)	2302 (18.5)	1128 (18.7)	1174 (18.32)	*p* = 0.448
Without disease	5703 (45.8)	3018 (50.0)	2685 (41.9)	*p* < 0.001
Presence of a single disease	2353 (18.9)	1172 (19.4)	1181 (18.4)	
Presence of Multimorbidity	4383 (35.2)	1842 (30.5)	2541 (39.6)	

**Table 3:** Distribution of health care utilization according to the presence of multimorbidity.

Variable	Category	Total	Absence of Multimorbidity	Presence of Multimorbidity	*p* *
Inpatient hospital care (past 12 months)	Yes	1051	370 (35.2%)	681 (64.8%)	<0.001
	No	11,379	7679 (67.5%)	3700 (32.5%)	
Day hospital (past 12 months)	Yes	852	317 (37.2%)	535 (62.8%)	<0.001
	No	11,571	7730 (66.8%)	3841 (33.2%)	
Use of physician-prescribed medications	Yes	5987	2289 (38.2%)	3698 (61.8%)	<0.001
	No	5792	5307 (91.6%)	485 (8.4%)	
Use of non-prescribed medications	Yes	4428	2445 (55.2%)	1983 (44.8%)	<0.001
	No	7345	5150 (70.1%)	2195 (29.9%)	
General practitioner or pediatrician (public sector)	Yes	11,043	6893 (62.4%)	4150 (37.6%)	<0.001
	No	1061	868 (81.8%)	193 (18.2%)	
General practitioner or pediatrician (private sector)	Yes	678	444 (65.5%)	234 (34.5%)	0.483
	No	11,440	7330 (64.1%)	4110 (35.9%)	
Gynecologist (public sector)	Yes	3448	2170 (62.9%)	1278 (37.1%)	<0.001
	No	2153	1227 (56.9%)	926 (43.1%)	
Gynecologist (private sector)	Yes	1058	808 (76.4%)	250 (23.6%)	<0.001
	No	4550	2594 (57.0%)	1956 (43.0%)	
Dentist (public sector)	Yes	3296	2142 (64.9%)	1154 (35.1%)	0.547
	No	8555	5610 (65.6%)	2945 (34.4%)	
Dentist (private sector)	Yes	5188	3746 (72.2%)	1442 (27.8%)	<0.001
	No	6684	4023 (60.2%)	2661 (39.8%)	
Last visit to a specialist physician	<12 months	5286	2559 (48.4%)	2727 (51.6%)	<0.001
	>12 months	5399	3926 (72.7%)	1473 (27.3%)	
	Never	1519	1384 (91.1%)	135 (8.9%)	
Visit to a physiotherapist (past 12 months)	Yes	1110	406 (36.6%)	704 (63.4%)	<0.001
	No	11,306	7635 (67.5%)	3671 (32.5%)	
Visit to a psychiatrist/psychologist (past 12 months)	Yes	610	241 (39.5%)	369 (60.5%)	<0.001
	No	11,803	7798 (66.1%)	4005 (33.9%)	
Use of private health care services (past 12 months)	Yes	3351	1952 (58.2%)	1399 (41.8%)	<0.001
	No	9074	6096 (67.2%)	2978 (32.8%)	
Use of home care services (past 12 months)	Yes	265	86 (32.4%)	179 (67.6%)	<0.001
	No	12,169	7966 (65.5%)	4230 (34.5%)	

* Chi-square test.

**Table 4:** Bivariate analysis and multivariate regression models of the association between demographic and socioeconomic characteristics and multimorbidity.

Variable	Category	Univariate Model OR (95% CI)	*p*	Multivariate Model OR (95% CI)	*p*
Sex	Female	1.495 (1.388–1.611)	<0.001	1.283 (1.172–1.404)	<0.001
	Male	1		1	
Age group (years)	20–29	0.016 (0.012–0.022)	<0.001	0.036 (0.025–0.052)	<0.001
	30–39	0.045 (0.036–0.057)	<0.001	0.102 (0.078–0.133)	<0.001
	40–49	0.105 (0.087–0.127)	<0.001	0.220 (0.174–0.278)	<0.001
	50–59	0.258 (0.217–0.308)	<0.001	0.486 (0.392–0.602)	<0.001
	60–69	0.554 (0.458–0.657)	<0.001	0.717 (0.596–0.863)	<0.001
	70–79	0.898 (0.748–1.079)	0.250	0.975 (0.807–1.178)	0.795
	80+	1		1	
Marital status	Single	0.166 (0.134–0.205)	<0.001	0.565 (0.442–0.723)	<0.001
	Married	0.915 (0.777–1.078)	0.290	0.835 (0.695–1.002)	0.154
	Widowed	2.856 (2.371–3.441)	<0.001	0.855 (0.688–1.061)	0.053
	Divorced	1		1	
Region	Vojvodina	1.261 (1.131–1.405)	<0.001	1.097 (0.964–1.248)	<0.001
	Šumadija and Central Serbia	0.910 (0.822–1.009)	0.073	0.756 (0.668–0.856)	0.162
	Southern and Eastern Serbia	1.364 (1.224–1.520)	<0.001	1.069 (0.934–1.223)	0.032
	Belgrade	1		1	
Education level	Primary or lower	3.493 (3.109–3.924)	<0.001	1.451 (1.252–1.681)	<0.001
	Secondary	1.203 (1.083–1.337)	0.001	1.096 (0.934–1.223)	0.155
	Higher/University	1		1	
Employment status	Unemployed	1.157 (0.759–1.764)	0.498	1.284 (0.807–2.044)	0.291
	Employed	4.578 (3.025–6.927)	<0.001	2.039 (1.287–3.229)	0.002
	Economically inactive	1		1	
Material status	Poor/Poorest	1.439 (1.324–1.563)	<0.001	1.216 (1.089–1.358)	<0.001
	Middle class	1.262 (1.141–1.397)	<0.001	1.109 (0.983–1.251)	0.093
	Rich/Richest	1		1	
Self-rated health	Poor/Very poor	1.733 (1.571–1.911)	<0.001	1.086 (0.973–1.211)	0.140
	Average	1.730 (1.555–1.924)	<0.001	1.295 (1.157–1.449)	<0.001
	Good/Very good	1		1	

**Table 5:** Bivariate analysis and multivariate regression models of the association between health care utilization and multimorbidity.

Variable	Category	Univariate Model OR (95% CI)	*p*	Multivariate Model OR (95% CI)	*p*
Inpatient hospital care	Yes	3.826 (3.351–4.368)	<0.001	1.455 (1.232–1.718)	<0.001
	No	1		1	
Day hospital	Yes	3.402 (2.945–3.929)	<0.001	1.430 (1.195–1.712)	<0.001
	No	1		1	
Use of physician-prescribed medications	Yes	7.628 (5.846–9.610)	<0.001	3.142 (1.741–4.709)	<0.001
	No	1		1	
Use of non-prescribed medications	Yes	1.902 (1.760–2.055)	<0.001	1.079 (0.981–1.188)	0.119
	No	1		1	
Last visit to a specialist physician	<12 months	10.892 (9.054–13.102)	<0.001	2.908 (2.340–3.614)	<0.001
	>12 months	3.846 (3.192–4.635)	<0.001	2.116 (1.708–2.622)	<0.001
	Never	1		1	
Visit to a physiotherapist (past 12 months)	Yes	3.581 (3.150–4.071)	<0.001	1.866 (1.591–2.190)	<0.001
	No	1		1	
Visit to a psychiatrist/psychologist (past 12 months)	Yes	2.986 (2.527–3.527)	<0.001	1.284 (1.042–1.583)	0.019
	No	1		1	
Use of private health care services (past 12 months)	Yes	1.465 (1.350–1.589)	<0.001	1.113 (1.001–1.238)	0.049
	No	1		1	
Use of home care services (past 12 months)	Yes	3.951 (3.047–5.123)	<0.001	1.460 (1.029–2.070)	0.034

## Data Availability

The original contributions presented in this study are included in the article. Further inquiries can be directed to the corresponding author.
